# Patterns of rapid weight loss in elite sambo athletes

**DOI:** 10.1186/s13102-021-00267-3

**Published:** 2021-04-14

**Authors:** Patrik Drid, Flavia Figlioli, Nemanja Lakicevic, Ambra Gentile, Valdemar Stajer, Bojan Raskovic, Nina Vojvodic, Roberto Roklicer, Tatjana Trivic, Sergey Tabakov, Sergey Eliseev, Antonino Bianco

**Affiliations:** 1 Faculty of Sport and Physical Education, University of Novi Sad, Lovcenska 16, Novi Sad, 21000 Serbia; 2 Sport and Exercise Sciences Research Unit, University of Palermo, Palermo, Italy; 3 Russian State University of Physical Education, Sport, Youth and Tourism, Moscow, Russia

**Keywords:** Weight control, Weight classes, Combat sports, Martial arts

## Abstract

**Background:**

Rapid weight loss (RWL) is commonly practiced in combat sports. Both magnitude and methods used to induce RWL are largely similar among combat sports, but currently, there is no data on RWL methodology used by sambo athletes. Therefore, the aim of this study was to determine RWL procedures sambo athletes apply to lose weight rapidly.

**Methods:**

The sample consisted of 199 participants, of which 132 males and 67 females who participated in the World Sambo Championship 2020 held in Novi Sad, Serbia. Each participant received RWL questionnaire that was available in multiple languages, and every participant was instructed how to fill it out.

**Results:**

Almost 87% of sambo participants declared to have intentionally cut their weight prior to the competition, whereby 5.27 kg (SD: ±7.57) was lost. Gradual dieting, sauna use and skipping meals were the most dominant methods used to reduce weight prior to competition while more extreme methods of RWL such as the use of laxatives, diuretics, diet pills and vomiting were also implemented but by much smaller fragment of the participants involved.

**Conclusions:**

Findings from our study largely match with previously conducted RWL studies in terms of prevalence, magnitude and methods used by combat sport athletes, especially in judo and wrestling. Knowing the hazardous consequences of RWL, alternative methods of sustainable weight loss should be considered.

## Background

Prior to 1940s the Committee of Sports of Union of Soviet Socialist Republics declared sambo to be the official combat sport of the Soviet Union [1]. In the decades to follow, sambo gained considerable attention in the post World War II Soviet Union states, but also on a global scale. After 80 years of its acknowledgment and development, sambo has recently received temporary recognition from the International Olympic Committee which is a first step towards inclusion in the Olympic Games [2].

Elite athletes are always aiming for a peak performance throughout the year. Sambo athletes are no different. Besides maintaining excellent physical shape and technical skills [3, 4], sambo athletes must maintain their optimal competitive weight for championships, given sambo is a weight-divided sport [2]. Thus, meticulous preparations (efficient training, adequate diet, sufficient sleep and recovery) are critical for every training session but especially for competitions. With regards to competitions and diet, studies reveal that nearly 90% of both male and female judo athletes engage in rapid weight loss (RWL) before the competition [5] to possibly gain competitive advantage over their lighter opponents. Similar trends have been observed in wrestling [6–8]. This approach of weight reduction is defined as a 5% weight loss achieved over 5–7 days [9, 10]. Regardless of the type of combat sport, methods of inducing RWL are very similar and are often initiated by reduced ingestion of fluids, caloric deficiency, increased training levels, plastic suit training, heated room training, and sauna use [7, 11–13]. Nevertheless, RWL procedure can cause many health complications that can affect the body acutely or chronically [14]. It is even more disturbing that some athletes declared performing RWL up to 10 times a year [5].

However, so far, no data has been obtained on RWL methods in sambo athletes. Therefore, the aim of this study was to identify the methodology and magnitude of RWL used by sambo athletes with a particular emphasis on sex-based differences.

## Methods

### Study design

To determine methods of RWL used by sambo athletes, we adopted RWL questionnaire developed by Artioli et al. [15]. The questionnaire consists of 21 items relating to personal information, competitive level, weight and diet history, and RWL behaviors, including the source of influence (e.g., teammates, coach, trainers, physician) and the methods used to cut weight before competition (e.g., vomiting, using pills, dieting, fasting, and so on) [15]. To ensure honest answers, we guaranteed that the questionnaire was totally anonymous. The original version of the questionnaire in Portuguese was used, and was further translated into Russian, French, Serbian, and Spanish language to facilitate data collection. Athletes were asked to fill out questionnaire in the sports hall where the competition took place. In case any questions were unclear, researchers were available to provide detailed explanation. Also, if any miscommunication was noted, a translator speaking all of the abovementioned languages was there to clarify details of the questionnaire.

### Participants

The sample consisted of 199 participants, of which 132 males (66.3%) and 67 females (33.7%). The mean age of the sample was 21.70 years (SD: ±5.22) for females and 23.16 years (SD: ±6.08) for males. The average height was 1.64 m (SD: ±0.09) for females and 1.75 m (SD: ±0.10) for males, while the average weight was 64.58 kg (SD: ±14.90) for females and 75.22 (SD: ±17.78) for males.

Participants from 20 countries took part in the study: Ukraine (20.1%), France (15.6%), Serbia (12.1%), Russia (11.1%), Moldavia (10.6%), Spain (7.5%), Uzbekistan (7.0%), Cameroon (3.0%), Bulgaria (2.5%), Kyrgyzstan (2.0%), Colombia (1.5%), Belarus (1.0%), Belgium (1.0%), Croatia (1.0%), Italy (1.0%), USA (1.0%), Lithuania (0.5%), Mexico (0.5%), South Africa (0.5%) and Tajikistan (0.5%).

The study was conducted according to the Helsinki declaration and ethical approval was obtained from ethics committee of University of Novi Sad, Serbia (Ref. No. 46–06-02/2020–1). All sambo athletes gave written informed consent upon agreement to participate. Since 30 participants were minor (younger than 18), written consent was obtained from their national team coach as their legal guardian during the championship.

### Statistical analysis

Obtained data were analyzed using SPSS statistical software (ver. 23.0). Descriptive statistics were first calculated on all the variables involved, including height, weight, athletic experience, RWL frequency, RWL methodology, and influence in weight-cutting practices. Gender differences regarding the amount of weight loss and regain were evaluated through t-test, while differences in RWL techniques and influences were calculated through the Wilcoxon Rank Sum test. The significance level was set at α = 0.05.

## Results

The average sambo experience of the participants was 11.37 years (SD: ±6.44). Most participants declared to have won at least one medal at international level (74.4%). The 86.9% (*n* = 173) of sambo participants declared to have intentionally cut their weight prior to the competition. In addition, athletes reported performing their first RWL at age 15.77 (SD: ±3.54). The sample declared to cut around 5.27 kg (SD: ±7.57) of body weight on average and usually started doing so 11.87 (SD: ±9.51) days before the competition (Table 1).

**Table 1:** Weight reduction history reported by the sambo competitors

Questions	Males		Females		***p***^**a**^
	Mean	SD	Mean	SD	
How many times did you cut weight to compete last season? (number of times)	4.41	8.35	3.51	3.03	0.43
How many days before competition do you usually cut weight? (start days)	10.97	9.88	12.04	9.10	0.50
At what age did you start to cut weight before the competition? (yrs.)	16.22	3.57	14.88	3.33	0.015*
	**Δ (%)**		**Δ (%)**		
How much weight do you usually cut before the competition? (kg)	−8.49		−5.59		0.15
What is the most weight you have cut to compete in your career? (kg)	−14.60		−13.30		0.80
How much weight do you usually regain after the competition? (kg)	11.70		6.42		0.03*

^a^ t-test comparison between male and female athletes

**Fig. 1:**
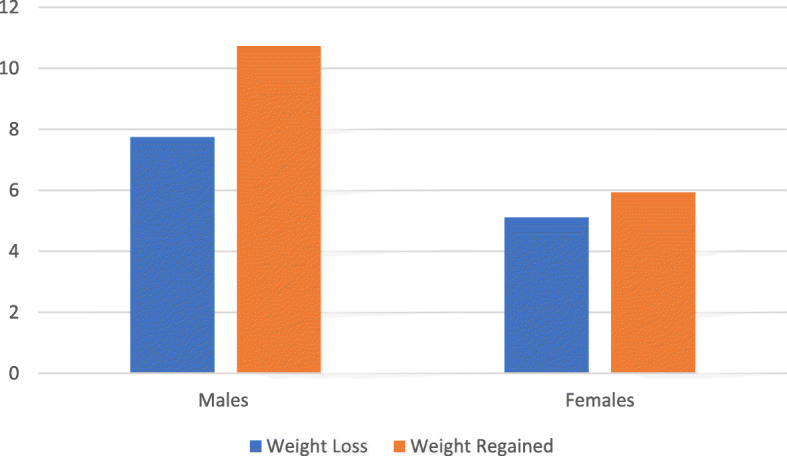
Percentage of weight loss and regained distinguished by gender (kg)

There were no significant gender differences in the amount of reduced weight, in both absolute and relative terms (Males: − 8.49%; Females: - 5.59%; Mean_males_: 5.82 kg, Mean_females_: 3.30 kg, *p =* 0.15,). Females started before males to cut weight prior to competition (Mean_females_: 14.88 days, Mean_males_: 16.22 days, *p* = 0.015) and tended to regain less weight than their male counterparts (Males: + 11.70%; Females: + 6.42%; Mean_females_: 3.83 kg, Mean_males_: 8.06 kg, *p* = 0.03) (Fig. 1).

The most common methods used to cut the weight rapidly (calculated as the sum of the answers “always” and “sometimes”) was gradual dieting (80.5%), followed by sauna (75.9%), skipping meals (70.7%), training with plastic suits (63.8%), not ingesting fluids (62.1%), training in a heated room (60.9%), increased exercise (58.0%), fasting (44.8%) and spitting (44.8%). Less common methods adopted were the use of laxatives (16.1%), diuretics (13.2%), diet pills (10.9%) and vomiting (9.8%) (Table 2).

**Table 2:** Frequency distribution for weight loss methods reported by the sambo competitors

Methods	I do not use it anymore	Never used	Rarely	Sometimes	Always
**Gradual dieting (%)**	**3.4**	**6.3**	**9.8**	**35.6**	**44.8**
Males	5.1	7.7	8.5	30.8	47.9
Females	0	3.5	12.3	45.6	38.6
**Skipping 1 or 2 meals (%)**	**2.9**	**10.3**	**16.1**	**53.4**	**17.2**
Males	3.4	11.1	17.9	51.3	16.2
Females	1.8	8.8	12.3	57.9	19.3
**Fasting (%)**	**8.0**	**30.5**	**16.7**	**37.9**	**6.9**
Males	7.7	32.5	21.4	32.5	6.0
Females	8.8	26.3	7.0	49.1	8.8
**Restricting fluid ingestion (%)**	**2.3**	**15.5**	**20.1**	**40.8**	**21.3**
Males	1.7	17.1	21.4	41.0	18.8
Females	3.5	12.3	17.5	40.4	26.3
**Increased exercise (%)**	**8.0**	**1.7**	**32.2**	**23.0**	**35.1**
Males	9.4	2.6	35.9	17.9	34.2
Females	5.3	0	24.6	33.3	36.8
**Training in heated room (%)**	**0.6**	**16.1**	**22.4**	**37.4**	**23.6**
Males	0.9	16.2	23.1	34.2	25.6
Females	0	15.8	21.1	43.9	19.3
**Sauna (%)**	**4.0**	**9.8**	**10.3**	**43.7**	**32.2**
Males	3.4	8.5	12.8	41.9	33.3
Females	5.3	12.3	5.3	47.4	29.8
**Training in plastic suits (%)**	**2.9**	**14.9**	**18.4**	**36.2**	**27.6**
Males	1.7	12.0	21.4	33.3	31.6
Females	5.3	21.1	12.3	42.1	19.3
**Use plastic suit all day (%)**	**2.9**	**40.2**	**19.5**	**23.6**	**13.8**
Males	0.9	39.3	23.1	21.4	15.4
Females	7.0	42.1	12.3	28.1	10.5
**Spitting (%)**	**3.4**	**36.2**	**15.5**	**36.2**	**8.6**
Males	3.4	31.6	14.5	41.9	8.5
Females	3.5	45.6	17.5	24.6	8.8
**Laxative (%)**	**5.2**	**67.8**	**10.9**	**13.2**	**2.9**
Males	1.7	68.4	12.0	14.5	3.4
Females	12.3	66.7	8.8	10.5	1.8
**Diuretics (%)**	**5.2**	**75.3**	**6.3**	**10.9**	**2.3**
Males	2.6	10.3	6.8	76.1	4.3
Females	1.8	12.3	5.3	73.3	7.0
**Diet pills (%)**	**2.9**	**79.3**	**6.9**	**6.9**	**4.0**
Males	1.7	78.6	8.5	6.8	4.3
Females	5.3	80.7	3.5	7.0	3.5
**Vomiting (%)**	**4.6**	**78.2**	**7.5**	**8.6**	**1.1**
Males	3.4	77.8	8.5	8.5	1.7
Females	7.0	78.9	5.3	8.8	0.0

With respect to gender differences in the methods used for cutting weight, Wilcoxon Rank Sum test showed non-significant outcomes for all the RWL methods.

Participants declared that they were very influenced or somehow influenced by their coach (60.1%). Physicians and dietitians were not influential for most athletes (58.4 and 62.6%, respectively) (Table 3). Wilcoxon Rank Sum test did not evidence any gender difference among the various figures of influence.

**Table 3:** Frequency distribution for the persons who are influential on the weight management behaviors reported by the sambo competitors

Source of influence	Not influential	Little influential	Unsure	Somehow influential	Very influential
**Teammate (%)**	**33.1**	**27.3%**	**5.8%**	**19.8**	**14.0**
Males	33.0	27.8	6.1	19.1	13.9
Females	33.3	26.3	5.3	21.1	14.0
**Fellow wrestler (%)**	**38.2**	**20.2**	**13.3**	**19.1**	**9.2**
Males	35.3	21.6	12.9	19.8	10.3
Females	43.9	17.5	14.0	17.5	7.0
**Physician (%)**	**58.4**	**19.7**	**6.9**	**10.4**	**4.6**
Males	54.7	17.9	9.4	14.5	3.4
Females	66.1	23.2	1.8	1.8	7.1
**Personal Trainer (%)**	**43.4**	**16.8**	**6.4**	**20.8**	**12.7**
Males	44.4	20.5	6.8	17.1	11.1
Females	41.1	8.9	5.4	28.6	16.1
**Coach (%)**	**16.3**	**14.5**	**8.7**	**28.5**	**32.0**
Males	17.2	16.4	11.2	24.1	31.0
Females	14.3	10.7	3.6	37.5	33.9
**Parents (%)**	**41.6**	**19.1**	**6.4**	**16.2**	**16.8**
Males	45.3	17.1	6.8	15.4	15.4
Females	33.9	23.2	5.4	17.9	19.6
**Dietitian (%)**	**62.6**	**13.2**	**8.0**	**9.8**	**6.3**
Males	57.3	17.1	8.5	10.3	6.8
Females	73.7	5.3	7.0	8.8	5.3

## Discussion

The aim of this study was to determine the methods sambo athletes use to induce RWL. Acquired data shows that gradual dieting, sauna use and skipping meals were the most common methods used to reduce weight prior to competition in both males and females. However, more extreme methods of RWL such as the use of laxatives, diuretics, diet pills and vomiting were not frequently used. The latter is especially worrisome not strictly because diuretics are prohibited by the World Anti-Doping Agency [16] and are responsible for a significant number of doping cases in combat sports [17], but because the devastating effects they can leave on one’s health [18]. Overall, males tended to lose more weight during RWL while also regaining more weight after RWL when compared to females. Findings from our study are largely similar to findings by Artioli et al. [5] where the most commonly used RWL methods by judoist were gradual dieting, increased exercising, skipping meals and restricted fluid ingestion. In addition, several studies on RWL methodology in high-school and college wrestlers show similar data [7, 19]. In other combat sports such as mixed martial arts, jujitsu, Brazilian jiu jitsu, boxing, taekwondo and kickboxing, prevalance of RWL ranges from 60 to 80% [11, 13, 20–22]. Therefore, detected RWL trends are not unique to sambo, judo or wrestling athletes but are omnipresent in varied degrees in combat sports in general.

Our study showed that coaches were the most important figure influencing sambo athletes to pursue RWL. Berkovich et al. [23] found identical results in his study on judo and taekwondo coaches and trainers. The same study showed that coaches and trainers encouraged athletes to engage in RWL as early as 12 years old. In our case, athletes reported starting RWL at ~ 16 years of age. This should come as no surprise since literature showed that even 5 year old wrestlers are pressured to engage in RWL [24]. The American Academy of Pediatrics stresses that this practice needs to be avoided at this age as it interferes with children’s and adolescents’ normal growth and development and can cause psychological issues [25].

Coming from a perspective that prioritizes athlete’s health and safety, it is important to outline that RWL practice is not without consequences. Existing literature eloquently describes RWL-induced physical and psychological consequences, which can range from acute to chronic [14], with repetitive RWL offering no protection for athletes from the negative impact of RWL on performance [26]. In fact, ever since 1996 eminent healthand fitness organizations such as the American College of Sports Medicine, have unambiguously stated that there is a general consensus that RWL has a negative impact on physiological and health-related parameters [27]. Despite these „early “warnings and some RWL-related regulations implemented in National Collegiate Athletic Association in United States [28], recent studies, including ours, reveal that tendencies regarding RWL in combat sports have not changed significantly [29]. It is important to outline that RWL is not necessarily associated with competitive success [30] or good performance [31, 32], and therefore the premise that lighter opponent will be likely the one to lose in the competition setting is questionable. However, oftentimes RWL is popularly perceived as a mental toughness practice that gives athletes a psychological advantage over their opponents [33]. Contrary to this belief, a recent systematic review on the impact of RWL on judo athletes by Lakicevic et al. [14] showed that the feelings of tension, anger, and fatigue significantly increased, while a decrease in vigor was demonstrated among judo athletes who practice RWL. As more and more emerging studies reveal harmful effects of RWL [9, 34–38] which can even lead to lethal consequences [39], scientists suggested that this harmful practice should be banned from combat sports [29]. Moreover, alternative means of weight loss such as gradual weight loss at the rate of about 1 kilogram per week, elevated carbohydrate intake and emphasis on weight loss from fat depots have been proposed [40]. Accordingly, strict regulations that will emphasize fairness and prioritize athlete’s health are needed to bring about much-needed change towards alleviation of RWL-induced consequences in combat sport athletes.

The current study comes with two main limitations: first, it has only an explorative purpose, therefore no causal inference has been done on the collected data. Moreover, questions about RWL could be sensitive to social desirability, thus participants could have been biased in their answers, even though we ensured that the questionnaire was totally anonymous.

Nevertheless, the study covers a topic that has never been addressed previously in sambo athletes and it was administered in people from more than 20 nations from all over the world. Since the majority of sambo athletes did not consult physician or dietitian about RWL, the results of the current study pertain primarily to coaches who should upgrade their knowledge about the risks of adopting RWL techniques, whereby athletes’ health will be the utmost priority.

## Conclusions

Nearly 87% of sambo athletes reported to have intentionally cut their weight prior to the competition, whereby 5.27 kg (SD: ±7.57) was lost. Gradual dieting, sauna use and skipping meals were the most dominant methods used to reduce weight prior to competition. Overall, males tended to lose more weight during RWL and regained more weight after RWL when compared to females. Findings from current study are consistent with results depicted in previously conducted RWL studies in terms of prevalence, magnitude and methods used by combat sport athletes, especially in judo and wrestling. Knowing the hazardous consequences of RWL, alternative methods of sustainable weight loss should be considered.

## Data Availability

The dataset used and/or analyzed during the current study are available from the corresponding author in response to a reasonable request. Due to patient’s data privacy data is not made available publicly.

## Abbreviation

RWL: Rapid Weight Loss
